# A multimodal pharmacological agent in combination with recanalization therapy (thrombolysis and thrombectomy) in severe stroke patients

**DOI:** 10.25122/jml-2019-1011

**Published:** 2019

**Authors:** Zdravka Poljakovic, Josip Ljevak, Svjetlana Supe, Katarina Starcevic

**Affiliations:** Department of Neurology, Intensive Care Unit, University Hospital, Zagreb, Croatia

**Keywords:** neurorecovery, stroke, recanalization, cerebrolysin

## Abstract

This case report highlights a case of large ischemic stroke and indication for anticoagulant therapy treated with thrombolysis and pharmacological intervention for neurological recovery with a multimodal agent (Cerebrolysin) as add-on therapy to recanalization techniques, including IV thrombolysis. We observed a significant clinical improvement after one year of follow-up. Based on our experience, we can assert that Cerebrolysin can be safely administered in stroke patients, even in complicated cases, with a good chance for improvement of their clinical status.

## Case Report

A 63-year-old male patient with a history of hypertension and cardiomyopathy was admitted to the emergency department due to sudden right-sided hemiparesis and speech disturbances. Symptoms started fifty-three minutes before the arrival at the hospital; the National Institutes of Health Stroke Scale (NIHSS) score at admission was 16. Urgent neuroimaging (native brain CT and CT angiography) showed a hyperdense middle cerebral artery (MCA) on the left side, without other brain parenchymal changes and occlusion of the M2 branch of the left MCA. Due to the eligibility of the patient, thrombolysis was started immediately, at 96 minutes after the onset of symptoms.

**Figure 1:**
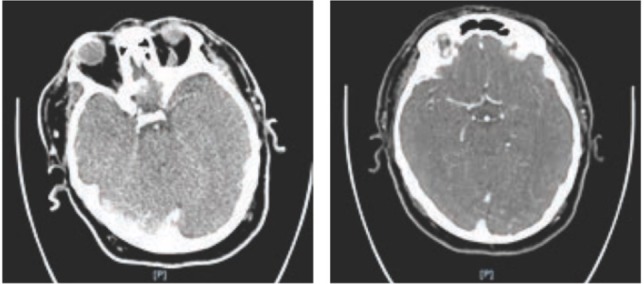
Native CT showing hyperdense left MCA, and CT angiography showing occlusion of the M2 branch of the left MCA.

However, after thrombolytic therapy, a minimal clinical effect was observed, with an NIHSS score of 14. Thrombectomy was indicated, and the procedure started after 2.5 hours after the onset of symptoms. Partial recanalization (2B grade on the Thrombolysis in Cerebral Infarction (TICI) scale) was achieved one hour later.

The NIHSS score after thrombectomy remained 14. A control CT scan after the procedures showed an acute ischemic lesion of the left hemisphere, including the insula, operculum, and caudal parts of the precentral and postcentral gyrus.

According to our institutional protocol, Cerebrolysin is indicated in this group of patients as early as possible, 24 hours after the symptom onset at the latest. Our patient started Cerebrolysin therapy (30 mL in 250 ml saline by intravenous infusion) 13 hours after symptoms onset with daily administration until discharge on day 27 [1-4].

On day 3, a neurorehabilitation program was initiated. During the hospitalization period, a urinary tract infection and deep venous thrombosis (despite correct prophylaxis) occurred as complications. Anticoagulant therapy was initiated five days before referral to the stationary rehabilitation on day 27. At discharge, the patient scored 4 points on the modified Rankin Scale (mRS) and 12 points on the NIHSS scale.

After 25 days of rehabilitation, the patient experienced a hemorrhagic shock due to rectorrhagia. He was admitted to the surgery ward for colectomy with a colostomy. By that time, before the surgery, his NIHSS score had improved to 8, with an mRS of 4. However, after the operation, at discharge, his general and neurological status worsened again. While the mRS score remained 4, from the perspective of the motor deficit, he deteriorated back to the status he had reached one month earlier. He was transferred back to the stationary rehab therapy for another ten days and received Cerebrolysin (30mL/day in 250 ml saline solution by intravenous infusion) again.

**Figure 2:**
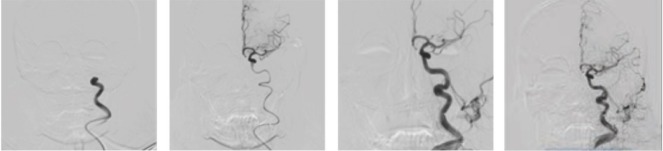
Angiography and thrombectomy, showing only partial recanalization (TICI 2B) of the ACM.

**Figure 3:**
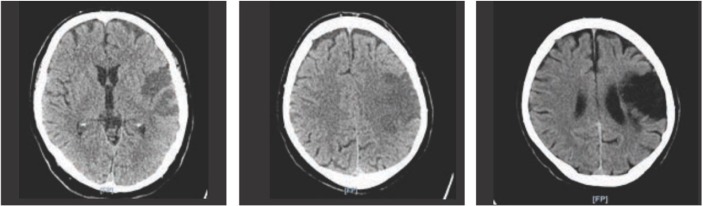
CT labeled A and B showing early ischaemic changes 24 hours after thrombectomy. CT labeled C was done at one-year follow-up.

Eight months after the stroke, he developed symptomatic focal epilepsy with impairment of consciousness and secondary generalization, which deteriorated his state to a greater extent. However, the patient continued with out-patient physical therapy. During our last out-patient visit, one year after the initial hospitalization, his mRS was 3 with an NIHSS score of 7, and he was strongly motivated to continue in his attempts for further improvement.

## Conclusion

Cerebrolysin, as add-on therapy to recanalization techniques, including IV thrombolysis, was safe in a patient with a large ischemic stroke and indication for anticoagulant therapy. In this particular case, we noticed a significant clinical improvement after one year of follow-up, despite less effective reperfusion (thrombolysis and thrombectomy) and complications resulting in anticoagulant therapy, surgical procedures, and symptomatic focal epilepsy. Cerebrolysin may have contributed to the favorable clinical outcome for our patient, in accordance with the neuroprotective and neurorestorative properties of Cerebrolysin [4-8]. Based on our experience, we can assert that Cerebrolysin can be safely administered in stroke patients, even in complicated cases, with a good chance for improvement of their clinical status.

## Conflict of Interest

The authors confirm that there are no conflicts of interest.
